# The Role of Cyclic AMP Signaling in Cardiac Fibrosis

**DOI:** 10.3390/cells9010069

**Published:** 2019-12-26

**Authors:** Marion Delaunay, Halima Osman, Simon Kaiser, Dario Diviani

**Affiliations:** Department of Biomedical Sciences, Faculty of Biology and Medicine, University of Lausanne, 1011 Lausanne, Switzerland

**Keywords:** cyclic AMP, protein kinase A, A-kinase anchoring protein (AKAP), adenylyl cyclase, phosphodiesterase, cardiac remodeling, cardiac fibrosis

## Abstract

Myocardial stress and injury invariably promote remodeling of the cardiac tissue, which is associated with cardiomyocyte death and development of fibrosis. The fibrotic process is initially triggered by the differentiation of resident cardiac fibroblasts into myofibroblasts. These activated fibroblasts display increased proliferative capacity and secrete large amounts of extracellular matrix. Uncontrolled myofibroblast activation can thus promote heart stiffness, cardiac dysfunction, arrhythmias, and progression to heart failure. Despite the well-established role of myofibroblasts in mediating cardiac disease, our current knowledge on how signaling pathways promoting fibrosis are regulated and coordinated in this cell type is largely incomplete. In this respect, cyclic adenosine monophosphate (cAMP) signaling acts as a major modulator of fibrotic responses activated in fibroblasts of injured or stressed hearts. In particular, accumulating evidence now suggests that upstream cAMP modulators including G protein-coupled receptors, adenylyl cyclases (ACs), and phosphodiesterases (PDEs); downstream cAMP effectors such as protein kinase A (PKA) and the guanine nucleotide exchange factor Epac; and cAMP signaling organizers such as A-kinase anchoring proteins (AKAPs) modulate a variety of fundamental cellular processes involved in myocardial fibrosis including myofibroblast differentiation, proliferation, collagen secretion, and invasiveness. The current review will discuss recent advances highlighting the role of cAMP and AKAP-mediated signaling in regulating pathophysiological responses controlling cardiac fibrosis.

## 1. Introduction

Heart failure is a chronic and lethal syndrome that leads to a progressive decrease in cardiac function. In the final stages of the disease cardiac output is no longer sufficient to match the oxygen and metabolic needs of the body, resulting in organ failure and death [1,2,3].

A number of stresses and insults can trigger heart failure including long-term hypertension, myocardial infarction (MI), excess production of hormones, and neurotransmitters, as well as exposure to drugs and toxicants [2,4,5]. Ventricular cardiomyocytes initially adapt to the increased workload imposed by these cardiac insults by hypertrophying [6,7]. While this compensatory process initially reduces the stress on ventricular walls and normalizes cardiac output, on the long term it predisposes to adverse ventricular remodeling associated with cardiomyocyte apoptosis, interstitial fibrosis, and impaired cardiac function [8,9,10]. 

It is now well appreciated that myocardial fibrosis represents a major cause of cardiac dysfunction in failing hearts. Accumulation of fibrotic tissue decreases myocardial compliance and affects electrical coupling between cardiomyocytes, reducing ventricular filling during diastole and causing arrhythmias, respectively [8]. 

During the last decade most of the translational research aiming at preserving and/or restoring cardiac function in failing hearts has focused on approaches targeting mainly cardiomyocytes [11]. Such strategies do not efficiently reduce fibrosis and associated cardiac dysfunctions, therefore the need of reorienting research on defining the key molecular mechanisms regulating cardiac fibrosis and on developing therapeutic tools targeting cardiac fibroblasts has become evident. In line with this assumption, recent findings demonstrate that targeting cardiac fibroblasts in stressed hearts using chimeric antigen receptor (CAR) T cells efficiently reduces fibrosis and cardiac dysfunction [12].

Accumulating evidence now indicates that cyclic adenosine monophosphate (cAMP) acts as major modulator of fibrotic responses. Indeed, cAMP-regulated signaling pathways have been shown to profoundly affect cardiac fibroblast function and impact the development of cardiac fibrosis [13,14]. In this context, the current review will highlight the main cellular mechanisms and molecular pathways contributing to cardiac fibrosis and discuss recent literature illustrating how cAMP signaling impacts pro-fibrotic responses in cardiac fibroblasts. The accent will be placed on individual regulators and effectors of cAMP signaling, on their implication in cardiac fibrosis, and on their role as potential targets for anti-fibrotic treatments.

## 2. Cellular and Molecular Mechanisms Controlling Cardiac Fibrosis

Cardiac fibroblasts represent the most abundant cell type in the myocardium and provide structural support through controlled proliferation and extracellular matrix (ECM) turnover [15]. In response to various cardiac stresses and insults such as MI and pressure overload, quiescent resident cardiac fibroblasts undergo transdifferentiation to myofibroblasts [16,17,18]. These activated fibroblasts display increased proliferatory and migratory properties, as well as enhanced ECM synthetic capacity, which allows them to colonize and remodel the injured myocardium. 

Cardiac myofibroblasts are characterized by the expression of α-smooth muscle actin (α-SMA) [15,19,20], which confers a contractile behavior to the cell, and they secrete large amounts of ECM proteins including pro-collagen I and III, periostin, and fibrillin [15,20]. Collagens are proteolyzed by matrix metalloproteinases (MMPs), subsequently cross-linked by lysyl oxidases and hydroxylases, and finally assembled into rigid and stable fibers, which promote heart stiffness, impair diastolic function, and contribute to heart failure [21]. 

Formation of myofibroblasts is induced by a variety of pro-fibrotic agonists including the transforming growth factor β (TGFβ), angiotensin II (Ang-II), aldosterone, and endothelin, as well as cytokines and chemokines produced in the injured or stressed myocardium [5,19,22,23]. These stimuli have been described to activate variety of pro-fibrotic intracellular signaling molecules such as Smad2/3 transcriptional regulators, the p38 mitogen activated protein kinase (MAPK), small molecular weight GTPases of the Rho family, and the transcription factor nuclear factor kappa B (NF-κB), which regulate gene programs associated with myofibroblast differentiation, proliferation, and migration, as well as ECM synthesis [22,24]. 

A large part of the scientific literature investigating the molecular mechanism of cardiac fibrosis is currently based on in vitro studies performed on neonatal or adult cardiac fibroblasts isolated from mice or rats, as well as on in vivo studies performed on global or cardiomyocyte-specific knockout mice. The inability to identify cardiac fibroblast-selective gene promoters that could drive transgenesis in this cell population has precluded, until recently, the possibility of studying the role of cardiac fibroblast-expressed signaling regulators in vivo. 

Recent studies have demonstrated that periostin, a secreted protein that becomes strongly upregulated during the process of cardiac myofibroblast differentiation, selectively marks this activated fibroblast population [20]. Based on this observation, transgenic mice harboring periostin promoter-driven Cre recombinase expression have been used to induce the selective KO of key pro-fibrotic regulators in cardiac myofibroblasts [20]. These studies have now confirmed the central role of TGFβ receptor-activated canonical (i.e., Smad2/3) and non-canonical (i.e., p38) pathways in the initiation and propagation of cardiac fibrosis in diseased hearts [22,24]. Strategies aiming at inhibiting these pathways in cardiac fibroblasts might therefore favorably impact cardiac function at late stages of heart failure. 

## 3. The Functional Role of cAMP Signaling in Cardiac Fibroblasts

**Figure 1:**
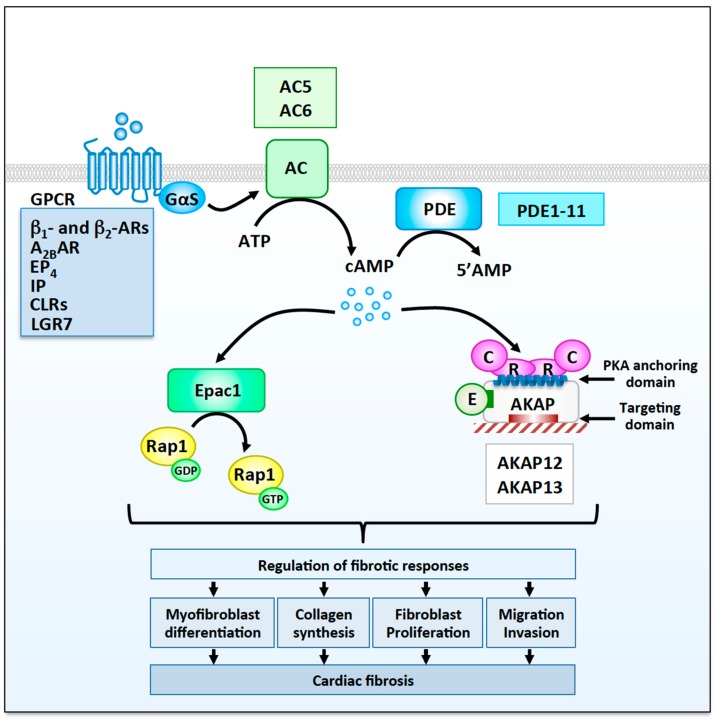
Cyclic adenosine monophosphate (cAMP) signaling modulators involved in the regulation fibrotic responses in cardiac fibroblasts. Cardiac fibroblasts express several Gs-coupled G protein-coupled receptors (GPCRs) including β_1_- and β_2_- adrenergic receptors (ARs), A_2B_ adenosine receptors (A_2B_Rs), prostaglandin E_2_ receptor 4 (EP4), prostacyclin receptors (IP), calcitonin receptor-like receptors (CLRs), and relaxin receptors (LGR7), which activate cAMP signaling cascades involved in the regulation of fibrotic responses. Stimulation of different membrane GPCRs is believed to induce the activation of distinct pool of ACs and the generation of separate cAMP microdomains. Phosphodiesterases (PDEs), which promote degradation of cAMP to AMP, are also involved in shaping intracellular cAMP microdomains. Two adenylyl cyclases, AC5 and AC6, as well as well as several phosphodiesterases families have been show to modulate cardiac fibrosis. cAMP activates two main effectors, Epac1 and protein kinase A (PKA). Epac1 catalyzes the GDP to GTP exchange on Rap1, whereas PKA, which are anchored at specific subcellular sites by A-kinase anchoring protein (AKAPs), promotes the phosphorylation of cellular substrates on serine and threonine residues. The anchoring sites for PKA and other signaling enzymes (E) as well as the AKAP targeting domains are indicated. Both Epac1 and PKA/AKAP complexes modulate, positively or negatively, multiple fibrotic responses including myofibroblast differentiation, collagen production, proliferation, migration, and invasion, which contribute to the development of myocardial fibrosis.

cAMP is a ubiquitous second messenger generated in response to neurohormonal stimulation of G protein-coupled receptors (GPCRs) linked to the stimulatory heterotrimeric G protein Gs. Ligand-activated GPCRs induce the exchange of GDP for GTP on the α subunit of Gs, thus promoting the release of active Gαs from the βγ dimer. This allows Gαs to activate adenylyl cyclases (AC), which, in turn, catalyze the formation of cAMP from ATP (Figure 1). Turnover of intracellular cAMP is ensured by phosphodiesterases (PDEs), which hydrolyze cAMP into inactive 5′AMP (Figure 1). There are four known cAMP effectors expressed in mammalian cells: the cAMP-dependent protein kinase (PKA) [25,26], the guanine nucleotide exchange factor Epac [27], the Popeye domain containing (Popdc) proteins [28], and the hyperpolarization cyclic nucleotide-gated (HCN) channels [29]. Among these effectors, only PKA and Epac exchange factors have been studied in cardiac fibroblasts. PKA is a broad-specificity basophilic serine/threonine kinase [25], whereas Epac proteins are guanine nucleotide exchange factors that specifically activate the small molecular weight GTPase Rap1 (Figure 1) [27].

To ensure that neurohormonal stimulation of membrane GPCRs is translated into an appropriate physiological response, cAMP signals are highly compartmentalized inside cells [30]. Firstly, localization of receptors and ACs at discrete plasma membrane domains favors local cAMP production [31]. Secondly, compartmentalization of PDEs further contributes in shaping cAMP microdomains by defining molecular barriers for cAMP diffusion [30,32]. Thirdly, organization of macromolecular signaling complexes containing ACs, PDEs, PKA, and its physiological substrates by a family of scaffolding proteins named A-kinase anchoring proteins (AKAPs), ensures spatiotemporal regulation of cAMP signaling events [31,33,34].

Early studies indicated that activation of intracellular cAMP signaling in isolated rat or mouse cardiac fibroblasts using forskolin (FSK) promotes anti-fibrotic effects as shown by a reduction in DNA synthesis, cell proliferation, and collagen production [13,35,36]. Similarly, incubation of cardiac fibroblasts with the membrane permeable cAMP analog 8-bromo-cAMP, which activates both PKA and Epac, impairs the profibrotic responses induced by AngII [36]. Subsequent studies taking advantage of cAMP derivatives that selectively activate PKA or Epac indicated that both signaling enzymes mediate the effects of cAMP on cardiac fibrosis [14]. More recently, the generation of knockout mouse models and the development of inhibitors targeting individual components of the cAMP transduction pathway has further improved our understanding of the functional relevance of cAMP signaling in cardiac fibroblasts. In this context, the following sections will summarize the current state of knowledge on the role of membrane GPCRs, cAMP regulators (ACs, PDEs), cAMP effectors (Epac, PKA), and cAMP signaling organizers (AKAPs) in transducing and shaping cAMP responses that influence cardiac fibroblast function and cardiac fibrosis.

### 3.1. G Protein-Coupled Receptors 

#### 3.1.1. β-Adrenergic Receptors 

β_1_- and β_2_- adrenergic receptors (β-ARs) are the main β-AR subtypes expressed in the human heart [37], with β_1_-ARs being predominant in cardiomyocytes and β_2_-ARs the most expressed subtype in cardiac fibroblasts [38]. While β_1_-ARs are mainly coupled to Gα_s_, β_2_-ARs can also couple to Gα_i_ upon phosphorylation of their third intracellular loop by PKA [39,40]. 

The role of β-ARs in cardiac fibrosis is currently matter of debate since studies performed over the last 15 years have led to conflicting conclusions. In this respect, β-ARs have been shown to promote both pro- and anti-fibrotic responses. On the one hand, stimulation of β_1_- and β_2_-ARs in isolated rat and human cardiac fibroblasts leads to increased DNA synthesis, proliferation, and production of pro-fibrotic interleukin 6 (IL-6) [41,42,43,44,45]. While β-AR-induced IL-6 release is mediated by cAMP and p38 MAPK pathways [46], proliferation appears to require the activation of cAMP-dependent pathways as well as epithelial growth factor (EGF) receptor transactivation [41,42]. On the other hand, β_2_-ARs also induce anti-fibrotic cAMP-dependent responses linked to the reduction of collagen synthesis and increase cardiac fibroblast autophagy, which are thought to be crucial for protecting failing hearts against excessive fibrosis induced by chronic exposure to catecholamines [47]. 

Most of the in vivo studies investigating the role of β-ARs in cardiac fibrosis rely on KO mouse models in which receptor expression is selectively suppressed in cardiomyocytes or in the entire animal. An important conclusion of these studies is that chronic stimulation of β_1_-ARs induces cardiomyocyte apoptosis whereas activation of β_2_-ARs promotes cardiomyocyte protection [48,49,50]. The differential impact of these two β-ARs subtypes on cardiomyocyte survival influences the occurrence and the extent of replacement fibrosis [50]. Unfortunately, however, since cardiac fibroblast-specific β-AR KO mice have not been generated so far, it is currently unknown whether and how β-ARs expressed in this cell type influence the development of cardiac fibrosis in injured or stressed hearts. 

Pharmacological therapies inhibiting β-ARs are currently used to treat heart failure because they reduce the ventricular afterload, decrease cardiac oxygen, and energy consumption, increase ventricular filling and improve patient survival. These effects are the result of the competitive inhibition of β-ARs expressed at the surface of cardiomyocytes. It is currently unknown whether blockade of β-AR affects fibrosis in humans. In this respect, experiments performed in animal models lead to puzzling results suggesting that β-blockers inhibit cardiac fibrosis in rats and promote fibrosis in mice hearts [51,52]. These inconsistencies might reflect differences in the expression of β-AR subtypes in cardiac fibroblasts or differential receptor coupling among species. 

#### 3.1.2. A_2B_ Adenosine Receptors

Adenosine is a ubiquitous cardioprotective signaling molecule that has been shown to play a central role in modulating cardiac remodeling and fibrosis following myocardial stress or damage [53]. It can be synthesized from extracellular cAMP through a two-step pathway that requires the metabolism of cAMP to AMP by ectophosphodiesterases and the conversion of AMP to adenosine by ectonucleotidases [54]. Among adenosine receptors expressed in the heart, A_2A_ and A_2B_ adenosine receptors (A_2A_Rs and A_2B_Rs) preferentially couple to Gs proteins [53]. The A_2B_R is the predominant adenosine receptor in cardiac fibroblasts, where it modulates multiple cellular responses contributing to cardiac fibrosis [53]. Studies performed on isolated neonatal and adult cardiac fibroblasts indicate that stimulation of endogenous A_2B_Rs with adenosine or A_2B_R-selective agonists efficiently inhibits proliferation, collagen synthesis and myofibroblast differentiation induced by pro-fibrotic agonists such as Ang-II, endothelin-1 (ET-1), TGFβ, and serum [54,55,56,57,58,59,60]. These effects appear to be mediated by intracellular cAMP signaling (Figure 1). In line with these observations, a study performed in rats indicates that infusion of stable adenosine analogs one-week post-MI significantly reduces myocardial fibrosis and attenuates cardiac dysfunction [61]. Moreover, recent evidence indicates that in mouse hearts subjected to pressure overload cardiomyocytes secrete cAMP, which after extracellular conversion to adenosine promotes anti-fibrotic effects by activating A_2_Rs at the surface of cardiac fibroblasts [62]. Altogether these findings suggest that A_2B_Rs mediated-cAMP signaling favors protection against cardiac fibrosis. 

In contrast to this conclusion, other studies suggest that knocking down A_2B_Rs or infusing A_2B_R selective antagonists in mice and rats subjected to MI or ischemia-reperfusion, protects against interstitial fibrosis and preserves ejection fraction (EF) and fractional shortening (FS), thus arguing for a pro-fibrotic effect of A_2B_Rs [53,63,64]. It should be emphasized, however, that these experimental approaches inhibit A_2B_R expression and activity not only in cardiac fibroblasts but also other cells populating the heart including cardiomyocytes, vascular smooth muscle cells, and immune cells infiltrating the damaged myocardium. Therefore, the observed phenotype might result from the global alteration of A_2B_R signaling in multiple cardiac cells. 

#### 3.1.3. Additional Gs-Coupled GPCR Regulating Fibrotic Responses 

In addition to β_2_ARs and A_2B_Rs, a few other Gs-coupled GPCRs including prostaglandin E_2_ receptor 4 (EP_4_), prostacyclin receptors (IP), calcitonin receptor-like receptors (CLRs), and relaxin receptors (LGR7) have been shown to inhibit cardiac fibrosis (Figure 1). These receptors display a dual anti-fibrotic effect. On the one hand, they can reduce cardiomyocyte death induced by MI or pressure overload and prevent replacement fibrosis and associated diastolic dysfunctions. On the other hand, they can activate multiple anti-fibrotic responses directly in cardiac fibroblasts. 

In particular, activation of EP_4_ and IP by synthetic analogs of prostaglandin E2 (PGE2) and prostacyclin, respectively, has been shown to inhibit TGFβ1-mediated synthesis of collagen type 1 and type 3 [65,66], and Ang-II-induced proliferation of rat cardiac fibroblasts [67]. These effects were shown to require cAMP production [65,67]. In line with these observations, KO of IP in mice increases cardiac fibrosis induced by Ang-II [65].

On the other hand, CLRs are crucial in mediating the anti-fibrotic effects of two peptide hormones, adrenomedullin (ADM), and intermedin (IMD) produced in heart. CLRs, unlike other GPCRs, need form stable complexes with receptor activity-modifying proteins (RAMPs) in order to bind their ligands. CLR-RAMP2 complexes respond to ADM whereas CLR-RAMP3 complexes are activated by IMD or its C-terminal fragment IMD 1-53 [68,69]. Interestingly, cardiac fibroblasts can synthesize and secrete ADM, which, via an autocrine/paracrine loop, inhibit fibroblast proliferation and collagen synthesis by increasing the intracellular cAMP concentration [70]. Similarly, it has been shown that IMD 1-53 significantly reduces Ang-II-induced cardiac fibroblast thymidine incorporation, collagen synthesis and myofibroblast differentiation through cAMP-dependent signaling [69]. 

Finally, LGR7 has been shown to mediate the anti-fibrotic effect of a peptide hormone named relaxin in isolated atrial and ventricular fibroblasts as well as in two mouse models of fibrotic cardiomyopathy [71]. Collectively, these findings suggest that Gs-coupled receptors expressed in cardiac fibroblasts are key modulators of cardiac fibrosis. Future research will need to focus on understanding the molecular mechanisms whereby cAMP modulated pathways activated by these receptors affect fibrosis in diseased hearts.

### 3.2. cAMP Regulators

#### 3.2.1. Adenylyl Cyclases

The AC family includes nine transmembrane isoforms and one soluble form [72,73]. They are characterized by a peculiar topological organization consisting of a cytosolic N-terminal domain, two cytoplasmic domains, C1 and C2, which form the catalytic core, and two regions containing six transmembrane helices [72]. 

Transmembrane ACs, except AC8, are expressed in the heart [74]. ACs 2–7 are detected in cardiac fibroblasts [75], while AC5 and 6 are predominantly expressed in cardiomyocytes where they control Ca^2+^ cycling and cardiac contractility [76]. ACs have been shown to be crucially involved in the regulation of pathological cardiac fibrosis and represent potential pharmacological targets in heart failure therapy. Initial evidence directly implicating ACs in anti-fibrotic responses comes from the observation that treating cardiac fibroblasts with FSK inhibits the pro-fibrotic effects of TGFβ and Ang-II and that overexpression of AC6 enhances the inhibitory effects of FSK on cardiac myofibroblast differentiation and collagen synthesis [13,35]. In adult rat cardiac fibroblasts, ACs can be targeted to focal adhesions through an interaction with phospho-caveolin1 [77]. At these sites it favors local cAMP production and activation of a PKA-dependent signaling pathway that inhibits actin cytoskeleton/focal adhesion assembly and myofibroblast differentiation [77]. Although not investigated, one can anticipate that AC-driven cytoskeletal disruption might also affect fibroblast migratory capacity. 

In vivo studies later showed that total AC expression and activity is significantly attenuated in cardiac fibroblasts isolated from rat hearts subjected to permanent ligation of the left descending coronary artery (LAD) [78]. Interestingly, reduced AC function correlated with increased collagen synthesis and secretion, thus arguing for an anti-fibrotic role for ACs expressed in heart fibroblasts [78]. 

Subsequent investigations revealed that AC5 and AC6, the two most abundant ACs in the heart, have opposite roles in regulating cardiac function. On the one hand, AC6 has been shown to exert cardioprotective and anti-fibrotic roles [79,80]. In particular, overexpression of AC6 in cardiac fibroblasts decreased collagen formation, myofibroblast transdifferentiation, and the expression of the pro-fibrotic genes induced by TGFβ [13]. In line with these results, a phase 2 clinical trial during which intracoronary gene transfer of AC6 was performed in heart failure patients highlighted clear beneficial effects of AC6 gene transfer on left ventricular function [81]. On the other hand, AC5 appears to contribute to aging-induced cardiomyopathy, as well as heart remodeling and dysfunction induced by chronic catecholamine stress or pressure-overload. Indeed AC5 KO mice are protected against hypertrophy, cardiomyocyte apoptosis, fibrosis, and heart failure [82,83,84]. The beneficial effects on cardiac function may result, at least in part, from the suppression of AC5 expression in cardiomyocytes [82,83]. 

During the last decade, substantial efforts have been made to develop selective inhibitors targeting AC5 over AC6 [85,86,87]. These studies led to the identification of C90, a potent AC5 inhibitor that has been shown to reduce infarct size in mice subjected to permanent LAD ligation with an IC50 of 30 nM [88]. A key advantage of C90 is that it maintains its protective action even when administered after coronary artery reperfusion. Importantly, C90 also decreases β-AR signaling, which might provide additional benefits to patients suffering from heart failure [88].

#### 3.2.2. Phosphodiesterases

Members of the PDE superfamily catalyze the hydrolysis of both cAMP and cGMP. There exist 11 PDE families with multiple isoforms characterized by distinct kinetic properties, substrate selectivity, and subcellular localization [89]. PDEs act as cyclic nucleotide “sinks” that shape cAMP and cGMP microdomains inside cells [32]. They are composed of a conserved catalytic region of approximately 270 amino acids and variable N-terminal et C-terminal regions ensuring differential regulation [90]. It is now demonstrated that PDEs can shape distinct cAMP microdomains controlling different biological functions [91,92]. The expression levels and the activity of several PDE family members is significantly altered in cardiac diseases associated with cardiac fibrosis including, MI, ischemia, and hypertrophic cardiomyopathies. Based on this evidence, pharmacological inhibition of PDEs is currently being considered as a potential therapeutic approach to treat these pathologies [90,92].

Among the PDEs expressed in cardiac tissues, PDE1, PDE2, and PDE10 families have been shown to directly modulate profibrotic functions in cardiac fibroblasts [91,93,94]. In particular, initial experiments performed on rodent and human failing hearts indicates that the expression of the PDE1A isoform is increased in cardiomyocytes and in fibroblasts of hearts undergoing cardiac remodeling [94]. Similarly, increased PDE1A levels are observed in isolated rat ventricular myocytes and fibroblasts stimulated by pro-fibrotic agonists such as TGFβ and Ang-II [91]. Importantly, inhibition of PDE1A protects mice from interstitial fibrosis induced by chronic isoproterenol treatment and reduces myofibroblast differentiation, pro-fibrotic gene induction, and matrix protein synthesis in cultured rat cardiac fibroblasts [94]. Fluorescence resonance energy transfer (FRET) studies indicate that PDE1 family members shape cAMP and cGMP microdomains localized in the perinuclear and nuclear area of cardiac fibroblasts, which may regulate pro-fibrotic gene programs [91]. Future studies will determine whether selective PDE1 inhibition can favor the regression of interstitial fibrosis and associated cardiac dysfunction in diseased hearts.

PDE2 has the capacity of hydrolyzing both cyclic nucleotides and is allosterically activated by cGMP [95]. It is encoded by the PDE2A gene and is upregulated in human and rat hearts suffering from ischemic, hypertrophic or dilated cardiomyopathies [96]. Transient overexpression of PDE2 in cardiac fibroblasts decreases the ability of β-ARs to induce cAMP synthesis and decrease myofibroblast differentiation induced by fibrotic agonists [93]. Engineered connective tissue generated from PDE2 overexpressing fibroblasts displayed increased rigidity as compared to control fibroblasts, suggesting that increased PDE levels promote excess ECM secretion [93]. Consistent with these findings, suppression of PDE2 activity in mouse hearts using the specific inhibitor BAY 60-7550 reduces cardiac remodeling and fibrosis induced by pressure overload [97]. Based on all these experimental evidences, pharmacological inhibition of PDEs is currently being considered as a potential therapeutic approach for the treatment of heart pathologies associated with cardiac fibrosis.

Recent evidence demonstrated that PDE10A, a dual specificity PDE capable of hydrolyzing both cAMP and cGMP, is strongly upregulated in failing hearts [98]. In vitro studies performed on isolated adult mouse cardiac fibroblasts, indicate that depletion or inhibition of PDE10A attenuates pro-fibrotic responses including cardiac fibroblast differentiation, proliferation, collagen synthesis and migration induced by TGFβ. Whole-body PDE10A KO inhibits Ang-II and pressure overload-induced cardiac hypertrophy, fibrosis and dysfunction, suggesting that pharmacological targeting of PDE10A might represent a potential strategy for the treatment of cardiac fibrosis and heart failure [98].

Finally, additional studies indicate that inhibition of PDE5 using sildenafil significantly reduces isoproterenol- and thoracic aortic constriction-induced expression of TGFβ, connective tissue growth factor (CTGF), collagen 1 and fibronectin 1 mRNAs in mouse hearts [99,100]. This correlated with reduced pathological cardiac hypertrophy and fibrosis. While these findings indicate that inhibiting PDE5 activity is cardioprotective, it remains to be established whether the anti-fibrotic effect of sildenafil are the consequence of PDE5 inhibition in cardiac fibroblasts. 

### 3.3. cAMP Effectors

#### 3.3.1. Exchange Protein Activated by cAMP 

The Epac family of guanine nucleotide exchange factors (GEFs) comprises two members encoded by independent genes, Epac1 and Epac2, which promote the activation of the small molecular weight G protein Rap1 [27,101]. At the structural level, they are characterized by an N-terminal regulatory region, which maintains Epac proteins in a basal autoinhibitory state, and a C-terminal catalytic region [102,103]. The N-terminal region of Epac1 and Epac2 contains a cyclic nucleotide binding (CNB) domain followed by a Dishevelled/Egl-10/pleckstrin (DEP) module, whereas a second CNB domain is present in Epac2 N-terminal to the DEP domain [103].

Binding of cAMP to the CNB sites induces a conformational change that relieves autoinhibition, which favors Epac activation and Rap1-GTP formation. Structure-function studies indicate that the DEP domain mediates the binding of Epac1 with the plasma membrane. 

Epac proteins have been observed at distinct subcellular compartments including plasma membrane, nuclear and mitochondrial membrane, in the cytosol surrounding the nucleus, and the cytoskeleton [104]. At these locations, Epac ensures spatio-temporal regulation of diverse cellular responses.

While Epac1 is ubiquitously expressed, Epac2 displays a more restrained tissue distribution and is mainly present in the brain and endocrine tissues [101]. In cardiovascular system, Epac1 is found in multiple cell types such as cardiomyocytes, endothelial cells, and cardiac fibroblasts [105,106,107]. Evidence accumulated over the last ten years indicates that Epac1 plays a crucial role in modulating several cardiomyocyte functions including contractility, action potential propagation, myofilament function, and cardiomyocyte adaptation to biomechanical or neurohumoral stress [105,108]. These findings have been reviewed extensively in recent years and will not be discussed further [109]. 

More recently, studies have started to investigate how Epac regulates fibrosis in stressed or diseased hearts. It appears that this exchange factor modulates both anti- and pro-fibrotic responses in cardiac fibroblasts. This underscores the complexity of Epac signaling and the difficulties that might be faced when targeting Epac proteins for the treatment of cardiac dysfunctions associated with myocardial fibrosis. The main findings illustrating these antithetical Epac functions are discussed below. 

Pro-fibrotic factors, including TGFβ1 and Ang-II, have been shown to decrease Epac1 mRNA expression in primary cultures cardiac fibroblasts. Epac1 is also downregulated after MI in cardiac fibroblasts from ventricles and atria [14,110]. Consistent with these results, Epac1 overexpression in cardiac fibroblasts inhibited TGFβ1-induced collagen secretion suggesting that a reduction in Epac1 levels may be necessary for initiating fibrotic responses [110]. Likewise, selective Epac1 activation in infarcted mouse hearts, following infusion of the hydrolysis-resistant cAMP analog sp-8-pCPT-2′-*O*-Me-cAMP, reduced cardiac dysfunction and diminished left atrial fibrosis [14]. However, it should be noticed that these anti-fibrotic effects could result from Epac1 inhibition in multiple cardiac cell types or be the consequence of a systemic effects induced by infused sp-8-pCPT-2′-*O*-Me-cAMP.

**Figure 2:**
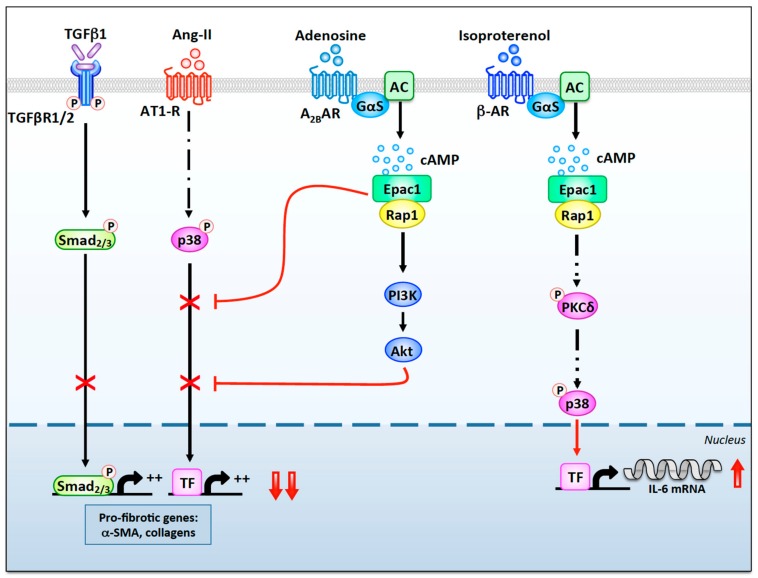
The anti- and pro-fibrotic roles of Epac1 in cardiac fibroblasts. Stimulation of A_2B_Rs by adenosine leads to the local activation of Gαs and AC (AC5 and/or AC6). Generation of cAMP enhances an Epac1-dependent anti-fibrotic pathway involving Rap1, PI3K, and Akt, which inhibits TGFβR1/2-Smad2/3 and AT1-R-p38 signaling. Epac1 can also inhibit Ang-II- and TGFβ1-induced pro-fibrotic signaling through a Rap1-independent pathway. In contrast, cAMP-mediated activation of Epac1 following β-AR stimulation leads to Rap1-dependent phosphorylation of PKCδ. Once activated, PKCδ translocates to the nuclear region where it activates p38, which enhances transcription of the IL-6 gene through the activation of an uncharacterized transcription factor (TF).

Epac1 also functions as a downstream effector of the anti-fibrotic pathways initiated by A_2B_Rs in cardiac fibroblasts. Indeed, stimulation of these receptors induces cAMP-dependent activation of Epac1, which, in turn, suppresses AngII- and TGFβ1-induced fibrotic responses (Figure 2) [58,59,60]. The inhibitory effects of Epac1 on collagen synthesis and α-SMA expression have been shown to require the activation of a Rap1-dependent pathway that includes the phosphatidylinositol 3-kinase (PI3K) and Akt, as well as a Rap1-independent signaling cascade (Figure 2) [58,59].

In contrast to the anti-fibrotic effects discussed above, Epac1 also enhances pro-fibrotic responses. Indeed, Epac1 overexpression in rat cardiac fibroblasts stimulates migration, a property known to be critical for myocardial invasion and a contributing factor for cardiac fibrosis development [110]. In line with these findings, subsequent studies performed on mouse cardiac fibroblasts demonstrated that Epac promotes the expression of pro-fibrotic IL-6 in response to the stimulation of β-ARs (Figure 2) [111]. Epac-mediated IL-6 production requires the sequential activation of PKCδ and p38 MAPK, which, in turn, stimulates IL-6 transcription (Figure 2) [111]. Future studies will need to assess whether these Epac-dependent signaling pathways are also activated in vivo. 

In vivo studies in mice point to a pathological and pro-fibrotic role for Epac1 in hearts undergoing remodeling. Indeed, whole-body Epac1 KO mice appear to be protected from cardiomyocyte hypertrophy, fibrosis, and cardiac dysfunction induced by pressure overload, ischemia reperfusion, and chronic β-adrenergic stimulation [112]. In contrast, mice displaying constitutively deletion of Epac2 show no protection against cardiac stress [112]. While these findings infer that Epac1-mediated signaling participate to heart failure, it is currently unknown whether reduction of cardiac fibrosis is the consequence deleting Epac1 in cardiac fibroblasts, cardiomyocyte, or in other cardiac cell types. Future studies using cardiac fibroblast and cardiomyocyte specific Epac1 KO mice will provide a more definitive answer to this question and clarify the role of Epac1 in cardiac fibrosis.

In recent years, development of selective Epac inhibitors and activators allowed an independent validation of the main findings obtained using Epac1 KO mice. While pharmacological inhibition of Epac1 reduces cardiac hypertrophy, fibrosis, inflammation and dysfunction induced by chronic stimulation of β-ARs [113], Epac activators such as 8-pCPT-2′-*O*-Me-cAMP and its esterified derivative have been shown to promote cardiac hypertrophy and fibrosis [114,115,116]. However, it should be noticed that these effects might also result form off-target effects due to the possible interaction of these activators with Epac2 and PDEs [114]. Recently, novel small molecule activators have been identified that displays greater selectivity towards Epac1 [117,118]. Their impact on cardiac remodeling and fibrosis still need to be tested. 

#### 3.3.2. Protein Kinase A 

PKA is tetrameric holoenzyme composed of a regulatory (R) subunit dimer and two catalytic subunits (C), which phosphorylate target substrates on the serine and threonine residues within the following consensus motifs: R-R-Φ-S/T or K-R-Φ-Φ-S/T (where Φ stands for hydrophobic residues) [25,119,120]. Binding of cAMP to the four binding sites located on the R subunit dimer induces the functional activation of the C subunit. In contrast to the classic view, which suggests that C subunits are released from the holoenzyme upon activation, recent evidence indicates that the catalytically active PKA remains intact (Figure 1) [121].

Four separate genes encode R subunits (RIα, RIβ, RIIα, or RIIβ) whereas there exist three different C subunit isoforms (Cα, Cβ, Cγ). One can differentiate two distinct PKA isoenzymes, which differ in their R subunit composition and subcellular localization: type I PKA is composed of RI subunits and is mainly cytosolic, whereas type II PKA contains RII subunits and is predominantly associated with specific cellular structures and organelles [122]. 

PKA has been shown to play key functions in cardiomyocytes, where it to regulates calcium cycling, myofiber contractility, cardiac repolarization, and the adaptive response to stress [123]. Surprisingly, while being abundantly expressed also in cardiac fibroblasts, its role in this cell type has been understudied in past years. 

Current literature suggests that this broad specificity kinase can either promote pro-fibrotic or anti-fibrotic responses. In particular, it has been shown that β1-ARs stimulate PKA activity to promote proliferation of cardiac fibroblasts [44], whereas activation of PKA by EP_4_ or IMD receptors suppresses DNA synthesis, collagen production, and myofibroblast differentiation induced by TGFβ [66,69]. One possible interpretation of these contrasting findings is that β1-ARs, EP_4_, and IMD receptors might induce the formation of distinct cAMP microdomains, which, in turn, would activate subcellular pools of PKA displaying different functions (i.e., pro-fibrotic or anti-fibrotic). In support of this hypothesis, β-ARs and EP_4_ receptors have been shown to be confined in separate membrane regions and induce distinct cAMP signals in cardiomyocytes [124,125,126,127].

**Figure 3:**
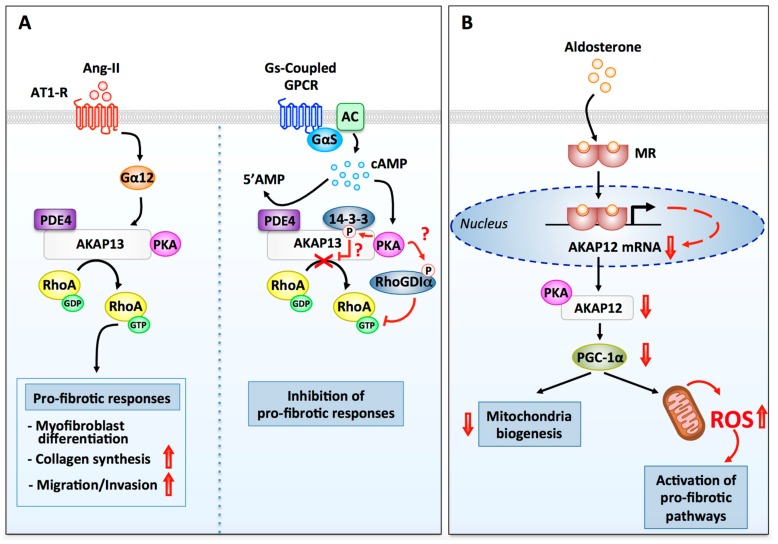
Regulation of fibrotic signaling pathways by PKA and AKAPs. (**A**) AKAP13-dependent modulation of fibrotic responses in ventricular fibroblasts. (Left side) Stimulation of AT1-Rs by Ang-II enhances AKAP13 Rho-guanine nucleotide exchange factor (GEF) activity through a signaling pathway that involves Gα_12_. Active RhoA released from AKAP13 promotes several pro-fibrotic responses including myofibroblast differentiation, collagen production, TGFβ1 production, migration, and invasion. (Right side) Phosphorylation of AKAP13 by anchored PKA is known to promote 14-3-3 recruitment and inhibition of AKAP13 RhoGEF activity. PKA has also been shown to phosphorylate Rho guanine nucleotide dissociation inhibitor α (RhoGDIα), which favors its association with RhoA-GTP and RhoA inhibition. It remains to be established whether AKAP13-anchored PKA can mediate these two inhibitory responses in cardiac fibroblasts. Termination of cAMP signaling is mediated AKAP13-anchored PDE4. (**B**) AKAP12-dependent regulation of reactive oxygen species (ROS) production in cardiac fibroblasts. Binding of aldosterone to mineralocorticoid receptors induces their nuclear translocation and the activation of a transcriptional response that results in the downregulation of AKAP12 mRNA and protein. Suppression of AKAP12 expression leads to the downregulation of PGC-1α. This inhibits mitochondrial biogenesis and promotes the production of ROS and oxidative stress, a potent activator of pro-fibrotic signaling pathways.

Activation of PKA in cardiac fibroblasts has also been shown inhibit the activity of RhoA, a small molecular weight GTPase which, by directly regulating actin cytoskeleton dynamics, controls cell morphology, migration, and adhesion. In particular, PKA phosphorylates the Rho guanine nucleotide dissociation inhibitor α (RhoGDIα) at serine 174, which increases its ability to interact with active RhoA and to sequester it in the cytosol (Figure 3) [128]. This inhibits RhoA-induced actin cytoskeleton dynamics and promotes cardiac fibroblast rounding. While not tested in this study, one can anticipate that disorganization of the actin network in rounded fibroblasts could also affect their ability to migrate (Figure 3). 

Collectively, it emerges that PKA plays a key role in modulating, positively or negatively, multiple fibrotic responses in cardiac fibroblasts. Subcellular targeting of distinct pools of the kinase to specific cellular substrates might influence the way PKA regulates fibrotic responses.

#### 3.3.3. A-Kinase Anchoring Proteins

AKAPs confer spatiotemporal regulation to PKA signaling [129,130]. This family of scaffolding proteins compartmentalizes PKA, upstream cAMP regulators (GPCRs, ACs, PDEs) and downstream effector substrates at precise subcellular locations to ensure that cAMP signals are processed locally and timely [34,130,131,132,133]. Interaction with PKA is mediated by conserved R subunit binding domains constituted by amphipathic helixes of 14–20 amino acids [134], which interact with the N-terminal dimerization/docking domain of the R subunits (Figure 1) [135,136]. While most of the AKAPs bind RII, a minority of anchoring proteins can interact with RI or with both regulatory subunits [137,138,139]. On the other hand, recruitment of signaling enzymes to AKAP signalosomes requires the presence of specialized protein-protein interaction motifs located on the anchoring protein (Figure 1). Finally, targeting of AKAP signaling complexes to specific cellular sites is mediated by anchoring domains that interact with compartment-specific proteins and lipids (Figure 1) [119,140]. 

In the heart, AKAPs have been shown to regulate multiple physiological responses including cardiac contraction, rhythm and energy production as well as pathological functions linked to the development of arrhythmias, cardiac hypertrophy, fibrosis, and heart failure [7,141,142]. Several studies indicate that altering AKAP expression and signaling in cardiomyocytes can significantly impact their ability to survive cardiac stress and consequently influence replacement fibrosis [33,140,143,144,145]. Since these results have been extensively reviewed in recent years, in the next paragraphs we will mainly focus on recent findings highlighting the role of AKAP signaling in cardiac fibroblasts. In particular, we will focus on two anchoring proteins, AKAP-Lbc and AKAP12, which have been shown to directly regulate fibrotic pathways.

##### AKAP13 

AKAP13 (also known as AKAP-Lbc) is a heart enriched anchoring protein expressed both in cardiomyocytes and cardiac fibroblasts [146,147]. This anchoring protein coordinates multiple signaling enzymes, such as PKA, protein kinase Cη (PKCη), protein kinase D1 (PKD1), PDE4D, phosphatases, and various MAPKs involved in the cardiac adaptation to stress and damage [148,149,150,151,152,153,154]. Unlike other AKAPs, AKAP13 also contains tandem Dbl homology (DH) and pleckstrin homology (PH) domains, which confer to the protein GEF activity towards RhoA and RhoC [146,155]. AKAP13 RhoGEF activity can be regulated bidirectionally by activating or inhibitory signals. Activation is mediated by the alpha subunit of the heterotrimeric G protein G12 [146], which interacts with a binding domain located in the C-terminus of the anchoring protein, whereas inhibition occurs following phosphorylation of serine 1565 on AKAP13 by anchored PKA, and subsequent recruitment of the regulatory protein 14-3-3 (Figure 3) [156,157]. 

While in cardiomyocytes AKAP13 activates RhoA and PKD1-dependent pathways involved in cardiomyocyte protection against stress [150,151,154], in cardiac fibroblasts it mediates profibrotic responses [147]. In this respect, experiments performed on adult rat cardiac fibroblasts revealed that AKAP13 silencing inhibits AT1-R-induced RhoA activation, collagen deposition, fibroblast migration, and differentiation of cardiac fibroblasts to myofibroblasts [147]. AT1-Rs enhance AKAP13 Rho-GEF activity through the activation of Gα12, whereas RhoA is the main profibrotic effector of AKAP13 (Figure 3) [147]. It is currently unknown how the AT1-R/Gα12/AKAP13/RhoA pathway initiates fibrosis. Based on previous findings showing that AKAP13 recruits and activates the MAPK p38 [151,158] and that p38 acts as a major mediator of the profibrotic effects of Ang-II [24], one could raise the hypothesis that AKAP13 might regulate cardiac fibrosis by promoting p38 signaling in cardiac fibroblasts.

The function of AKAP13-bound PKA in cardiac fibrosis is yet to be defined. However, knowing that this kinase can inhibit AKAP13-RhoGEF activity and enhance RhoGDIα-RhoA interaction in cardiac fibroblasts, one could speculate that the pool of PKA anchored to AKAP13 might be involved in the activation of anti-fibrotic responses leading to the inhibition of RhoA signaling (Figure 3). 

##### AKAP12

AKAP12 (also known as gravin, AKAP250, or SSeCKS) is expressed in various cell types of the cardiovascular system including vascular smooth muscle and endothelial cells, cardiomyocytes, and cardiac fibroblasts [159,160,161,162]. In addition of interacting with PKA, AKAP12 also associates with β-ARs, PKC, polo-line kinase 1, calmodulin, and PDE4D [163,164,165,166,167,168]. In mouse hearts, AKAP12 expression is strongly downregulated in response to chronic exposure to profibrotic agonists such as Ang-II [169]. This could suggest that AKAP12 downregulation might be linked to the development of cardiac fibrosis. In line with this assumption, whole body AKAP12 KO exacerbates cardiomyocyte dysfunction, apoptosis, and interstitial fibrosis induced by Ang-II infusion [169]. Enhanced fibrosis was associated with an increased number of α-SMA expressing cells in the myocardium. 

These observations, which suggest an anti-fibrotic role for AKAP12, were further validated by in vitro studies investigating the pro-fibrotic effect of mineralocorticoid hormones on primary cultures of human cardiac fibroblasts. Aldosterone, the primary mineralocorticoid in mammalians, can be produced in the myocardium in response to cardiac stress and insults and contributes to the development of cardiac fibrosis and dysfunction [170]. Interestingly, sustained aldosterone stimulation of human cardiac fibroblasts induces AKAP12 downregulation with a concomitant reduction of the expression of the peroxisome proliferator-activated receptor γ coactivator 1α (PGC-1α), a transcriptional regulator crucially involved in mitochondrial biogenesis (Figure 3) [159]. This leads to an impairment of mitochondria biogenesis, which promotes cardiac oxidative stress. Consistent with these results, knockdown of AKAP12 in human cardiac fibroblasts mimics the deleterious effects of aldosterone, whereas over-expression of the anchoring protein inhibited aldosterone-induced mitochondrial dysfunction and reactive oxygen species (ROS) production (Figure 3) [159]. Since oxidative stress is a major activator of fibrotic pathways in cardiac fibroblast, one could suggest that downregulation of AKAP12 might represent a mechanism whereby aldosterone promotes cardiac fibrosis (Figure 3).

Interestingly, AKAP12 to has also been shown to play a central role in the resolution of hepatic fibrosis [171], raising the possibility that AKAP12 might function as an anti-fibrotic regulator in multiple organs. 

## 4. Conclusions and Perspectives

Cardiac fibroblasts play a central homeostatic role by determining, through controlled ECM synthesis and degradation, the structural cohesion of the entire heart. In the injured heart, activated fibroblasts are crucially involved in initiating the wound-healing process. However, uncontrolled or excess activation of cardiac fibroblasts may result in interstitial fibrosis, which contributes to severe diastolic dysfunctions, arrhythmias and heart failure [172]. In past years, research investigating the molecular determinants of heart failure has focused on cardiomyocytes, considering this cell type as the main determinant of cardiac function. It has since become evident that events occurring in fibroblasts significantly contribute to development of heart failure. In this context, it emerged that cAMP signaling plays a central role in regulating cardiac fibroblast function both in healthy and diseased hearts. In particular, investigations performed on isolated cardiac fibroblasts as well as in vivo studies carried out in whole-body KO mice subjected to various cardiac stresses or insults revealed the implication of upstream cAMP modulators (GPCRs, ACs, PDEs), downstream cAMP effectors (Epac1, PKA) and cAMP signaling coordinators (AKAPs) in the regulation fibrotic process. While in vitro studies were instrumental in deciphering the molecular mechanisms and transduction pathways whereby cAMP affects individual fibrotic responses (i.e., myofibroblast differentiation, proliferation, collagen production, interleukin synthesis, migration and invasion) in cardiac fibroblasts, unfortunately the studies performed on constitutive KO animals do not allow to demonstrate a direct link between alterations of cAMP signaling in cardiac fibroblasts and the development of cardiac fibrosis. 

In this respect, future investigations will be able to take advantage of recently developed transgenic mouse models harboring collagen 1a (Col1a), Tcf21 or periostin promoter-driven Cre-recombinase expression to knockout cAMP regulators, effectors and AKAPs in resident cardiac fibroblasts or cardiac myofibroblasts, respectively [20,173] Cardiac fibroblast-specific promoters could be also used to overexpress peptides disrupting the interaction between AKAPs and pro-fibrotic effector proteins to selectively inhibit fibrotic responses.

Subcellular compartmentalization of cAMP signaling proteins including receptors, ACs, PDEs, Epac, and PKA ensures that signals generated by membrane GPCRs is timely translated into specific physiological responses [92]. Appropriate positioning of membrane GPCRs, ACs, and PDEs is central for generating and shaping spatially distinct cAMP microdomains that regulate diverse cellular functions [92]. While these concepts have been widely demonstrated in cardiomyocytes, so far, it is currently unknown whether cAMP microdomains generated in cardiac fibroblasts contribute to the fibrotic process. Future studies using FRET based-approaches might be able to visualize cAMP microdomains and determine which ACs and PDEs contribute to shape them. 

While several AKAPs are expressed in cardiac fibroblasts, so far only a few studies have addressed their role in cardiac fibrosis. Given the central role of this family of anchoring proteins in organizing macromolecular signaling complexes that coordinate the activity of cAMP signaling regulators and effectors one could anticipate that AKAPs might be key in specifying the effects of cAMP on cardiac fibrosis. In depth, molecular characterization of the AKAP signaling complexes expressed in cardiac fibroblasts could allow the identification of key protein-protein interactions that control fibrotic signals. Such protein interfaces could be targeted by peptides or small molecule compounds to inhibit cardiac fibrosis. In this respect, two studies recently identified small molecules inhibiting the ability of AKAP13 to bind and activate RhoA [155,174]. It will be crucial to determine whether such molecules can efficiently inhibit the pro-fibrotic function of the AKAP-Lbc/RhoA pathway both in vitro and in vivo. 

In conclusion, based on all the evidence accumulated so far, it emerges that development of experimental approaches enhancing activity of anti-fibrotic cAMP effectors and inhibiting signaling through pro-fibrotic cAMP complexes might represent a potential strategy for reducing fibrosis in hearts subjected to stress.
